# Chromosomal origin of replication coordinates logically distinct types of bacterial genetic regulation

**DOI:** 10.1038/s41540-020-0124-1

**Published:** 2020-02-17

**Authors:** Kosmas Kosmidis, Kim Philipp Jablonski, Georgi Muskhelishvili, Marc-Thorsten Hütt

**Affiliations:** 1 Division of Theoretical Physics, Physics Department, Aristotle University of Thessaloniki, 54124 Thessaloniki, Greece; 2 PharmaInformatics Unit, Research Center ATHENA, Athens, Greece; 3 Department of Life Sciences and Chemistry, Jacobs University, Bremen, Germany; 4 Department of Biology, Agricultural University of Georgia, Tbilisi, Georgia; 5 Present Address: Department of Biosystems Science and Engineering, ETH Zürich, Zürich, Switzerland

**Keywords:** Genetic interaction, Computational biology and bioinformatics, Regulatory networks

## Abstract

For a long time it has been hypothesized that bacterial gene regulation involves an intricate interplay of the transcriptional regulatory network (TRN) and the spatial organization of genes in the chromosome. Here we explore this hypothesis both on a structural and on a functional level. On the structural level, we study the TRN as a spatially embedded network. On the functional level, we analyze gene expression patterns from a network perspective (“digital control”), as well as from the perspective of the spatial organization of the chromosome (“analog control”). Our structural analysis reveals the outstanding relevance of the symmetry axis defined by the origin (Ori) and terminus (Ter) of replication for the network embedding and, thus, suggests the co-evolution of two regulatory infrastructures, namely the transcriptional regulatory network and the spatial arrangement of genes on the chromosome, to optimize the cross-talk between two fundamental biological processes: genomic expression and replication. This observation is confirmed by the functional analysis based on the differential gene expression patterns of more than 4000 pairs of microarray and RNA-Seq datasets for *E. coli* from the Colombos Database using complex network and machine learning methods. This large-scale analysis supports the notion that two logically distinct types of genetic control are cooperating to regulate gene expression in a complementary manner. Moreover, we find that the position of the gene relative to the Ori is a feature of very high predictive value for gene expression, indicating that the Ori–Ter symmetry axis coordinates the action of distinct genetic control mechanisms.

## Introduction

In spite of the tremendous progress made in Systems Biology^1–3^ and the construction of computational models of biological cells,^4,5^ we still lack the appropriate understanding of the underlying principles of genetic regulation to predict, for example, the gene expression pattern of a bacterium. Since the beginning of Systems Biology, the investigation of bacterial gene regulation has been an important source of hypotheses about the principles of biological regulation.^6–9^ The transcriptional regulatory network (TRN) of the classical model organism *Escherichia coli* has been the subject of a vast number of statistical analyses. In fact, this network has been the first example of a complex network for which a non-random network motif distribution (deviations from randomness of the counts of small subgraphs) has been reported.^6,10^ In spite of its prominence and the diversity of investigations, this network has been mostly studied in isolation.

It is becoming ever clearer that beyond network topology itself, the spatial embedding of complex networks provides an important additional layer of information for understanding a network’s function.^11,12^ While this aspect has been explored in transportation networks,^13,14^ brain networks^15,16^ and a wide range of other natural and technical systems,^17,18^ it has not been studied in much detail in the gene regulatory system. In particular, only few aspects of the spatial embedding of the *E. coli* TRN have been studied before, e.g., the spatial (i.e., chromosomal) distribution of genes with and without a reported link in the TRN^19,20^ and the orientation of genes on the genome.^21^

It is also intuitive (and in fact a prominent research trend of the last years, see e.g.,^12^) that spatially embedded networks need to be analyzed with a different set of tools than graphs without such a spatial embedding. For example, the concept of a dimension, which has been rarely discussed in complex network theory was found to be an important property of spatially embedded networks.^22^ In a spatially embedded network typically long-ranging links have a different systemic purpose than short-ranging links. For example, in social networks most people have their friends in their neighborhood, and the arrangement of connections in power grids and transportation networks obviously depends on the distance between the connected units. Considering the network of passenger flights, it is systemically plausible that such links occur only above a certain spatial distance. The transcriptional regulatory network is a somewhat non-standard example of a spatially embedded network, as the “space”, i.e., the 3D organization of the circular chromosome, is not immediately obvious. We explore the hypothesis that bacterial gene regulation is organized as an interplay of two distinct types of control—one exerted by the TRN (“digital control”) and one arising from the spatial organization of the chromosome (“analog control”). This hypothesis has been formulated,^23,24^ put into a broader context^25,26^ and supported by statistical analyses^27–29^ in a range of studies over the last decade, but has yet to be confirmed as a consistent organizational principle across all layers of quantitatively assessable information.

**Fig. 1:**
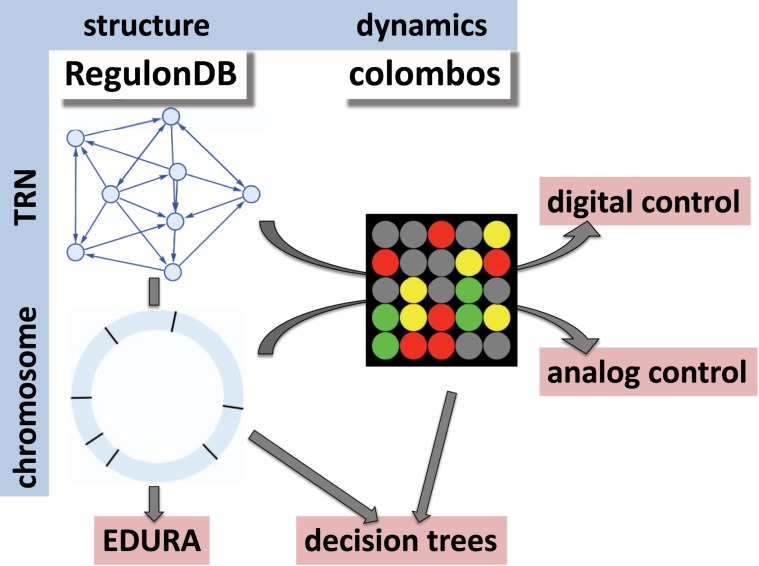
Summary of our investigation and overview of the workflow. On the structural level (obtained from RegulonDB,^55^), the spatial embedding of the TRN within the circular chromosome is evaluated via the EDURA (Edge Distribution Under Rotation of an Axis) method. The functional level is contributed by the COLOMBOS database^30^ and analyzed jointly with the structural information using the concepts of digital and analog control strengths,^27^ as well as decision trees.

**Fig. 2:**
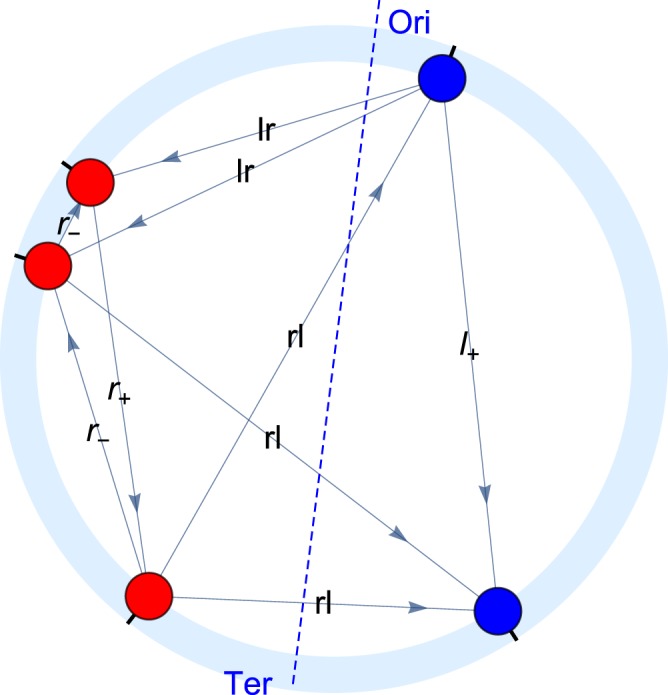
Illustration of the edge categories used in the subsequent analysis. The light blue circle represents the circular chromosome, while the dots represent genes (red: right chromosomal arm; blue: left chromosomal arm). Directed edges indicate interactions between genes. The dashed blue line denotes the axes used for the assignment of edge categories (with the longer end representing Ori).

Here we first address the interplay of digital and analog control on a purely structural level, by analyzing the chromosomal embedding of the TRN of *E. coli*. Next, we extend this investigation to a functional level by employing a method proposed in the ref. ^27^ to quantify the strengths of the two control types by using data from the COLOMBOS^30^ database and perform a large-scale study of the interplay between digital and analog control. COLOMBOS is a collection of expression data from published microarrays and RNA-Seq experiments performed in *E. coli* (and several other prokaryotes). COLOMBOS combines expression data analyses across different research papers, labs, and platforms. A key idea in COLOMBOS is to compare relative expression values to a reference state as sets of “condition contrasts”. This should correct for platform-dependent differences between studies. The expression data contained within the database are also linked to a manually curated, standardized condition annotation, and ontology. Figures 1, 2 summarizes the design of our study.

By analyzing (i) the distribution statistics of links in the transcriptional regulatory network, (ii) the agreement of gene expression patterns with both, the TRN and the distribution of genes in the genome, and (iii) the “learnability” of gene expression patterns by a decision tree employing various chromosomal and regulatory features we have been able to establish two main components of the logic underlying bacterial gene expression: (1) The Ori–Ter axis is a relevant organizer of gene expression; (2) Chromosomal structure (“analog control”) and transcription factors (“digital control”) contribute to regulation in an “either–or” fashion with one level of control buffering the other.

## Results

### Structural evidence for the interplay of digital and analog control and the relevance of the Ori–Ter axis

We start by employing methods from statistical physics of complex networks, in order to identify the non-random features characterizing the chromosomal embedding of the transcriptional regulatory network.

A key assumption of our investigation is that in spite of the complex and spatiotemporally variable 3D organization of the *E. coli* chromosome, the linear organization given by the positional order of genes along the chromosome is a relevant coordinate system for investigating a non-random spatial embedding of the TRN. This assumption is strongly supported by the study of distributions of genes with and without TRN participation,^20^ the statistical analysis of gene expression patterns along the chromosomal coordinates^27,31^ and the phenotypic changes contingent on positional shifts of genes encoding the transcription factors in the chromosome.^32,33^

Perhaps unsurprisingly, application of standard tools for the analysis of spatially embedded networks does not reveal striking non-random features of the chromosomal embedding of the TRN and only provides weak evidence for the co-evolution of these two biological structures^34^ (see Supplementary Figs 1, 2).

As a consequence, we develop and apply a set of methods tailor-made for the biological system analyzed here, thus addressing the core question: Is the transcriptional regulatory network systematically shaped by the chromosomal embedding?

Given the known relevance of the Ori–Ter axis dividing the circular chromosome into the right and left arms for genetic regulation,^31^ we can rephrase the question: Is the chromosomal embedding of the transcriptional regulatory network particular with respect to the Ori–Ter axis? Our analysis strategy in the following is to compute network quantities under rotation of this axis and see, whether the true axis stands out. The method, termed EDURA (edge distribution under rotation of an axis; see Fig. 2) is based on the principles described below.

Given the position of an axis, we distinguish between six categories of edges, namely edges on the right chromosomal arm pointing away from the origin of replication Ori, *r*_+_, or towards the Ori, *r*_−_ and the same on the left arm, *l*_+_ and *l*_−_, respectively, as well as edges across the two chromosomal arms, from right to left, *rl*, and from left to right, *lr*. Figure 1 illustrates these categories using a small sample graph. The labels “left” and “right” are understood looking from Ori to Ter. A schematic representation of the EDURA method is given in Supplementary Fig. 3.

**Fig. 3:**
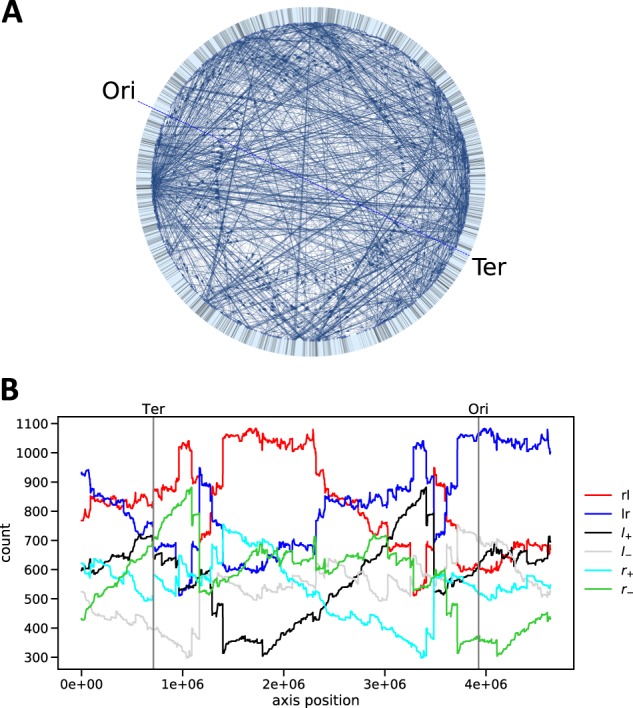
Analysis of edge categories in the *E. coli* TRN. **a** Representation of the chromosomally embedded *E. coli* TRN. As in Fig. 1 the large light blue circle represents the circular chromosome and the blue line indicates the Ori–Ter axis. Black dashes on the chromosome indicate genes. **b** Edge categories for the chromosomally embedded *E. coli* TRN from **a** as a function of the axis position. The true Ori and Ter positions are indicated as a reference.

It is clear that, when a different axis is chosen, these edge categories change. The counts *n*(*r*_+_), etc. can now be evaluated for each position of an axis. Under rotation of the axis an edge will undergo a systematic sequence of category transitions. Starting from *lr* as an example, a typical sequence under axis rotation will be *lr* → *r*_+_ → *rl* → *l*_−_. As a consequence, counts of link types are highly correlated and strongly dependent on gene density and node degree. Figure 3 shows the counts *n*(*r*_+_), etc. as a function of the axis position for the real chromosomally embedded *E. coli* TRN. The highly volatile nature of these counts, as well as the strong influence of gene density, systematic transitions (leading to high correlations among the curves) and contribution of hubs (e.g., dramatic changes in the curves due to many edges changing category at the same time) are clearly visible.

In order to remove these direct effects on the edge categories, we have to (1) consider asymmetries, rather than absolute counts, of the edge categories and (2) subtract the average signal observed in a randomized graph (see Methods section). The ± asymmetry indicating a mismatch between edges going down and going up for one chromosomal arm can be defined as

1 $${A}_{\pm }^{r}=\frac{n({r}_{+})-n({r}_{-})}{n({r}_{+})+n({r}_{-})+n(rl)},$$

and accordingly for ${A}_{\pm }^{l}$. The “cross–along” asymmetry, measuring the asymmetry between edges along the chromosomal arms and across them, is defined as

2 $${A}_{\leftrightarrow \updownarrow }=\frac{n(lr)+n(rl)-n({l}_{+})-n({l}_{-})-n({r}_{+})-n({r}_{-})}{n(lr)+n(rl)+n({l}_{+})+n({l}_{-})+n({r}_{+})+n({r}_{-})},$$

which is the number of edges across the arms minus the number of edges along the arms, normalized by the total number of edges. Without any clear systematics with respect to a given axis, the asymmetries will display strong correlations, due to the transition rules outlined above. Any disruption of these correlations at some axis position is an indicator of the non-random features of the network for this axis.

**Fig. 4:**
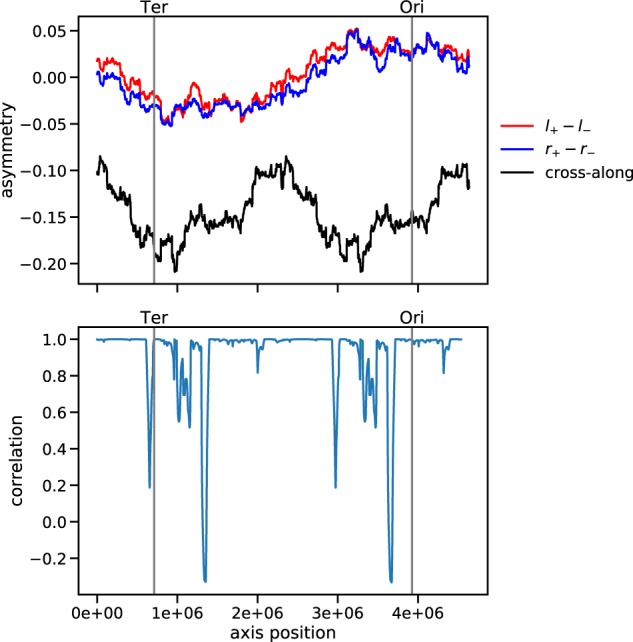
Edge category asymmetry analysis for the real *E. coli* TRN. Asymmetries as a function of the assumed axis position (upper panel). Correlation coefficient of ${A}_{\pm }^{r}$ and ${A}_{\pm }^{l}$ as a function of the assumed axis position (lower panel).

Using randomly generated networks with a systematic edge distribution with respect to an axis (systematic random networks; see Methods section) we can test and calibrate the EDURA method (see Supplementary Information). These tests show that, indeed, a specific axis inscribed in the edge distribution is detected via the EDURA method as drastic drops in correlation among the edge category asymmetries for this axis position (see Supplementary Fig. 4). In Fig. 4 the same analysis is performed for the real *E. coli* TRN.

The first statistical analysis supports the earlier findings,^20^ where via point process statistics it was found that genes under known direct transcriptional regulation are systematically more distal on the chromosome than genes without transcriptional regulation, confirming the general idea that bacterial gene regulation is organized as an interplay of network-based (digital) control and (analog) control based on the spatial organization of the chromosome. In order to go beyond the confirmation of the previous finding^20^ we resort to a common technique of network science, the comparison with randomized graphs as a method for identifying the higher-order non-random features of a given network. In this way we find structural evidence for the chromosomal embedding of the network being systematic with respect to one characteristic spatial axis in the circular chromosome (Fig. 4). This axis is defined by the origin (Ori) and terminus (Ter) of replication. Previously it was argued that the spatiotemporal organization of genomic transcription indeed follows the two replichores as the main spatial organizers.^31^ Here we find that the network architecture itself carries an evolutionary imprint of this bilinear space defined by the two replichores.

### Functional evidence for the interplay of digital and analog control

In order to assess the functional implications of this structural interdependence of the transcriptional regulatory network and the spatial organization of the chromosome we resort to the COLOMBOS representation of the Gene Expression Omnibus (GEO) database. For a large number of gene expression datasets, we measure the agreement of significant expression changes with the network and with chromosomal neighborhoods by employing the quantification methods for digital and analog control strengths defined previously.^27^

We start our investigation by creating the transcriptional regulatory network (TRN) and the gene proximity network (GPN) of the *E.coli* genome. Details of these two networks are found in the Methods section.

Then, for each of the ~4000 experimental datasets obtained from the COLOMBOS database we generate “effective” TRN and GPN networks by removing the nodes that are not significantly differentially expressed as well as all their links.

Supplementary Fig. 5 (left) shows and example of such an “effective TRN”. It depicts data from the experiment GSE10158 which contains microarray data on the expression profile of *E. coli* treated with cefsulodin and mecillinam, both alone and in combination. The figure shows the 22 genes whose expression level was significantly altered comparing the contrasts with id’s GSM256904_ch1 and GSM256868_ch1. Genes on the graph are positioned on a circle according to their coordinates on the *E.coli* chromosome. Supplementary Fig. 5 (right) presents a view of an “extended” TRN subgraph which contains the differentially expressed genes (blue points) plus all the genes that are connected to the differentially expressed ones in the *E. coli* TRN although without significantly altered expression levels (yellow points). The extended network comprises 90 genes. A complete understanding of regulatory control, about which the present manuscript is a first step, should aim in explaining why the “blue” genes were differentially expressed while the “yellow” ones were not, despite their immediate connection on the TRN which indicates a strong interaction between the two.

**Fig. 5:**
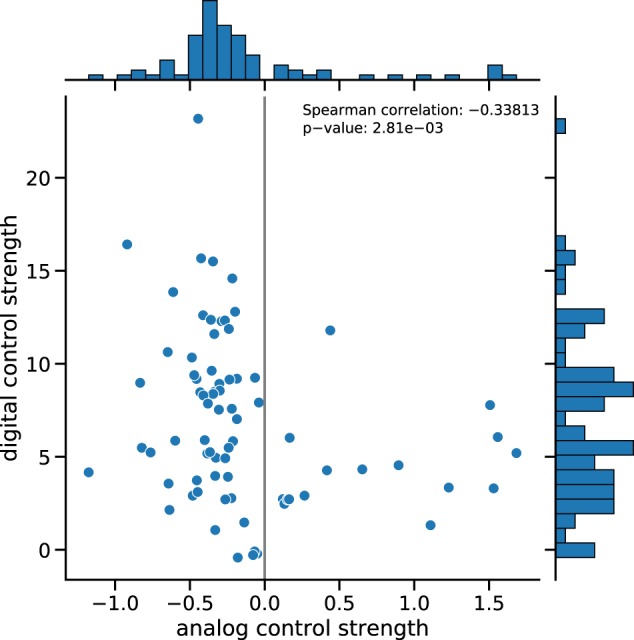
Digital vs. analog control for gene-level RNA-Seq data. Data for 104 effective networks resulting from contrasts of RNA-Seq experiments have been analyzed. Central panel: Scatter plot of the digital vs. analog control strengths. Top panel: Histogram of the distribution of analog control strength. Right panel: Histogram of the distribution of digital control strength.

**Fig. 6:**
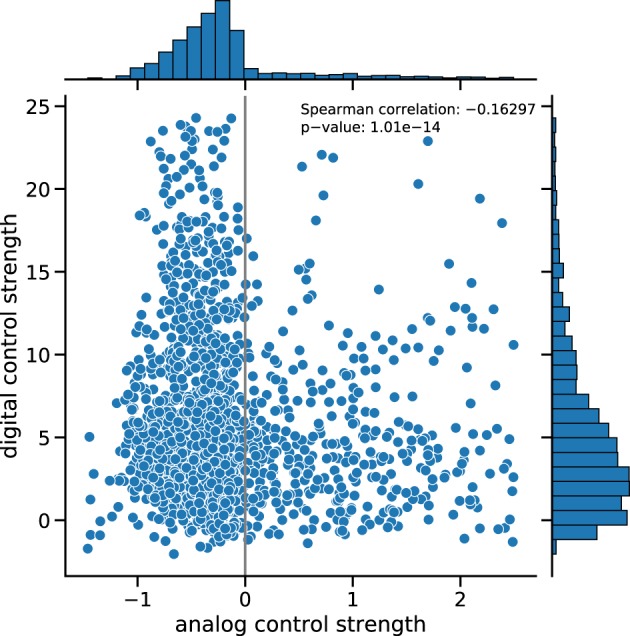
Digital vs. analog control for gene-level microarray data. Data for 3969 effective networks resulting from contrasts of microarray experiments have been analyzed. Central panel: Scatter plot of the digital vs. analog control strengths. Top panel: Histogram of the distribution of analog control strength. Right panel: Histogram of the distribution of digital control strength.

Figures 5, 6 show scatter plots of the digital vs. analog control strengths of more than 4000 effective *E. coli* networks derived from the COLOMBOS database (see Methods section and particularly the Effective Networks subsection). Figure 5 shows data for 104 high quality RNA-Seq experiments. The results demonstrate an anti-correlation between digital and analog control strength with a Spearman correlation coefficient of −0.34.

Figure 6 shows data for 3969 effective networks constructed from contrasts of microarray experiments. The results demonstrate once more an anti-correlation between digital and analog control strengths with a Spearman correlation coefficient of −0.16. A heatmap representation of the corresponding rank-based scatterplots is given in Supplementary Figs 15 (RNA-Seq) and 16 (microarray). In line with the negative correlations discussed here, these heatmap representations show the buffering relationship between digital and analog control: high rank values of analog control go along with low rank values of digital control and vice versa.

Our analysis reveals the systematic anti-correlation of digital and analog control as a large global trend in bacterial transcriptomes. In this way, it confirms the buffering of these two categories of bacterial gene regulation that was hypothesized before^27^ based on a set of transcriptome profiles obtained under a dedicated experimental variation of both, the machinery of digital control (via the analysis of hub mutants in the transcriptional regulatory network) and analog control (via the alteration of supercoiling energy in the genome induced by topoisomerase poisons). Our new results show that the anti-correlation of these distinct logical types of regulation is not limited to perturbations of the regulatory machinery, but persists across a wide range of experimental conditions (phenotypes) and genotypes.

Strikingly, the anti-correlation between digital and analog control strengths is much stronger for significantly downregulated than for upregulated genes, when these two categories are analyzed separately (see Supplementary Figs 8, 9). This statistical difference between downregulated and upregulated genes is in line with the earlier observation^35^ that the “ground state” (or default state) in prokaryotic gene regulation is nonrestrictive (or “on”), as opposed to eukaryotic gene regulation, where the ground state is restrictive (or “off”). It is then intuitive that the systematic interplay between digital and analog control reveals itself in the pattern of deviations from the ground state, i.e., in the downregulated genes.

We have re-computed our results varying the two main parameters of our analysis, namely the logFC threshold for determining differentially expressed genes and the distance threshold defining links in the GPN (see Supplementary Figs 11, 12). This parameter variation confirms the robustness and strong statistical validity of our findings. As an additional confirmation we computed the interplay of digital and analog control strengths also on the operon level (see Supplementary Figs 13, 14), leading qualitatively to the same results.

Summarizing, our results demonstrate for the first time that the tight coupling between digital (network-based) and analog (chromosomal) control goes far beyond the response to perturbations specifically designed to affect one of the two control types (as reported previously^27^), but is a universal property of bacterial gene regulation.

### Decision tree analysis of transcriptome profiles and the relevance of the Ori–Ter axis

In order to ascertain that the anti-correlation observed in the large set of transcriptome profiles is not dependent on our choice of quantification method, namely the digital and analog control strengths, we also used a machine learning framework in order to measure, which structural features are employed to predict significant expression changes from the database of transcriptome profiles, when the feature set consists of nine quantitative variables (see Methods section, Decision Trees) selected to represent a wide range of network and chromosomal properties. This set of features is specifically designed to highlight the impacts of either digital or analog control in a given expression profile.

Here we are not interested in the quality of the classification (and, hence, do not separate the data into training and test data), but rather in the features employed by the decision tree to split the genes in an expression contrast into “differentially expressed” and “not differentially expressed”. As our main goal is to assess the interplay between digital and analog control at work in these gene expression patterns, we use a set of features, which can either be associated with digital control (number of differentially expressed regulators of a gene in the TRN, hns regulating the gene, fis regulating the gene, crp regulating the gene) or analog control (number of differentially expressed neighbors in the GPN, hns binding site density near a gene’s location, fis binding site density, crp binding site density), as well as one feature not directly classifiable as digital or analog control, namely the position of a gene relative to the Ori. For each gene expression contrast we can now compute the relative importance of each of these features in classifying genes according to their differential expression. The question here is, whether the decision tree predominantly employs analog features in contrasts with high analog control strength and, conversely, digital features in contrasts with high digital control strength.

In this analysis, we use the binding sites only as a proxy for local structural features of chromosome. Previous studies demonstrated that the gene order along the Ori–Ter axis is highly conserved in bacteria.^31^ This spatial order is apparent not only for the principal regulatory genes (such as e.g., RNA polymerase sigma factors and nucleoid-associated proteins) but also for their targets. Furthermore, while the chromosomal position of a gene is thought to be determinative for the gene copy number and expression level,^36–39^ recent studies strongly suggest that it is also determinative for the spatial location of the gene product in the cell. In particular, the regulatory proteins were found to diffuse from their cognate sites of production forming gradients.^40,41^ This suggests that the genomic distances between the transcriptional regulators and their targets are subject to evolutionary constraints and that in general, regulatory genes would be preferentially positioned in the vicinity of target genes.^21^ However, spatial considerations imply a different effect in the case of highly abundant DNA architectural proteins, such as e.g., the nucleoid-associated proteins, that diffuse over relatively large distances, bind cooperatively at hundreds of chromosomal sites and compact the DNA by constraining DNA supercoils over extended chromosomal regions.^24,42–44^ Variable spatial distributions of nucleoprotein complexes formed by global regulators such as e.g., FIS and H-NS modulate the structural dynamics of the chromosome exerting continuous or analog effects on genetic expression reflected in directionally coherent changes of transcript patterns involving neighboring genes^45,46^ that can be readily measured by estimating the analog control strength in the effective networks.

We have generated decision trees for each of the microarray and RNA-Seq effective networks in our possession and used them to estimate the importance of all of the nine features in each case. In order to exclude the effects of randomness in the estimation of importance, the differentially expressed genes for each of the individual effective networks were shuffled 100 times and decision trees were used to estimate the feature importance of these randomized cases. Subsequently, we subtracted the mean of the randomized importances from the actual feature importance.

**Fig. 7:**
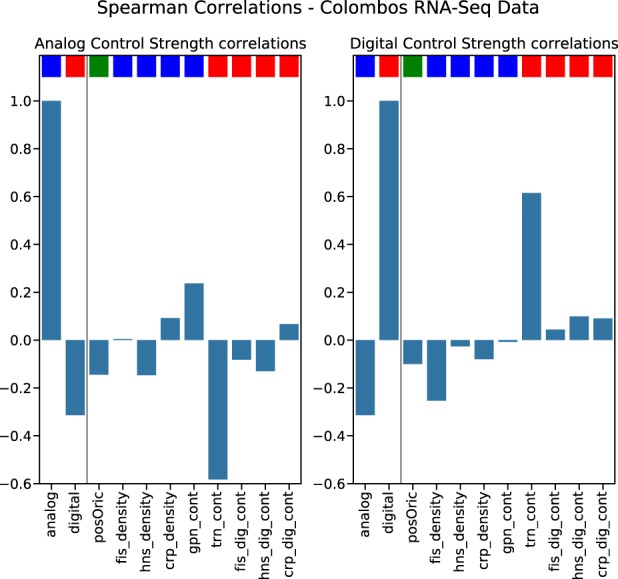
Feature importance correlations for RNA-Seq data. (Left) Spearman correlation coefficient between analogCTC and each of the features for networks derived from the rnaseq experiments. (Right) The same for the digitalCTC. The color code above each bar is: blue—analog control feature, red—digital control feature, green—dual feature (related to the Ori–Ter axis).

**Fig. 8:**
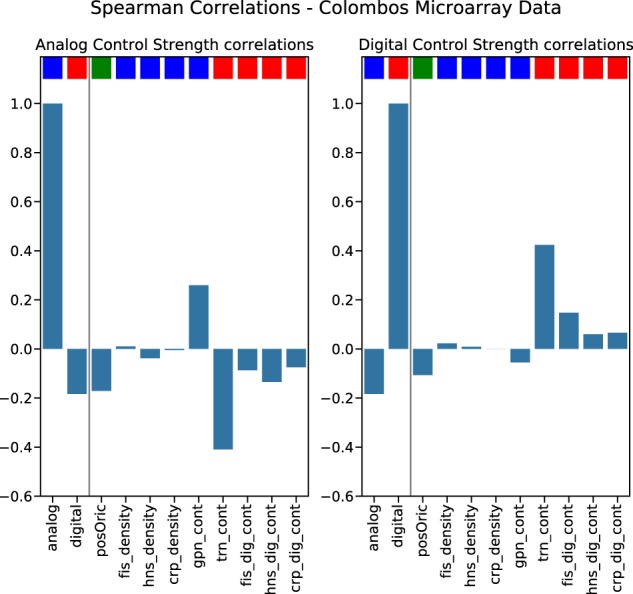
Feature importance correlations microarray data. (Left) Spearman correlation coefficient between analogCTC and each of the features for networks derived from the microarray experiments. (Right) The same for the digitalCTC. The color code above each bar is: blue—analog control feature, red—digital control feature, green—dual feature (related to the Ori–Ter axis).

The results form a matrix of nine columns and 3969 rows for the microarray data and 104 rows for the RNA-Seq data. This matrix can be augmented, if we include the digitalCTC and the analogCTC as two additional columns, especially since we are interested in investigating how the proposed measures of digitalCTC and analogCTC correlate with the features used to characterize the expressed *E. coli* genes. As before, the RNA-Seq and microarray experiments were analyzed separately. Figure 7 (left panel) shows the Spearman correlation coefficient between analogCTC and each of the features for networks derived from the RNA-Seq experiments. Figure 7 (right panel) shows the same, but for the digitalCTC. Similarly, Fig. 8 (left panel) shows the Spearman correlation coefficient between analogCTC and each of the features for networks derived from the microarray experiments and Fig. 8 (right panel) shows the same for the digitalCTC.

In all cases we observe a high correlation between digitalCTC and the “digital” features, i.e., *trn cont*, *hns dig cont*, *fis dig cont* and *crp dig cont*. We also observe a high correlation between analogCTC and *gpn cont,* which is a very characteristic analog control feature. The overall trend is in agreement of what is expected by the assumption of the existence of two complementary forms of transcriptional control and provides a proof that the quantities of analogCTC and digitalCTC are a reliable means of measuring the impact of digital and analog control mechanisms of gene regulation in the expression profiles.

The systematic anti-correlation between features associated with digital control and features associated with analog control, as well as the relevance of the Ori–Ter axis (via the relevance of the distance from Ori as a feature integrated in the machine learning analysis) confirm the results from the previous two sections.

More specifically, we find that the relative position of a gene with regard to the chromosomal Ori, that is along the Ori–Ter axis, is a feature of very high predictive value for gene expression. Despite its clear impact, we cannot readily attribute the influence of the Ori–Ter symmetry axis either to purely digital or to purely analog type of control. Therefore, we consider this axis as a third essential and distinct regulatory element of the system, i.e., a coordinator of gene expression.

## Discussion

In this study we set out to integrate two different modes of transcriptional regulation, one mediated by the TRN and another by spatial organization of the chromosome. For this purpose we applied a set of structural and functional analyses. We hypothesized that the TRN is spatially embedded in the chromosome such that the pattern of directed links is highly non-random with respect to a single axis converting the circular space (i.e., the circular chromosome) into two linear branches (the chromosomal “arms”). We set out to identify this organizational principle, as well as the position of the axis, by computing a set of statistical quantifiers for each candidate position of such an axis and then observing clear systematics in the behavior of these topological features emerging when we approach the true axis position. This novel data analysis is called EDURA (Edge Distribution Under Rotation of an Axis). One counter-intuitive aspect of the EDURA result is that the disruption of correlations is the relevant signal. This follows from the transformation properties of link categories indicated in the previous section with *l*_+_ transforming first into *rl* and then into *r*_−_ upon rotation of the axis. Highly correlated link counts are therefore the default, while a sudden drop in correlation is indicative of a systematic arrangement of links with respect to this position of the axis. We have tested and confirmed this view by a detailed analysis of random graphs with a systematic link distribution bias with respect to a randomly selected axis (see Methods section, “Systematic random networks”; see Supplementary Fig. 4 for an example of EDURA for a systematic random network). Beyond the set of findings on bacterial gene regulation, we are convinced that the EDURA method, together with the axis-systematic random networks, will be of relevance for the analysis of a range of other spatially embedded networks.

We discovered a systematic orientation of the network with respect to the Ori–Ter axis indicative of an evolutionary co-adaptation of replication and transcriptional regulation. Indeed, in *E. coli* and other bacteria the collisions between the transcription machinery and the replisomes progressing bi-directionally from the Ori towards the Ter pose problems potentially leading to genetic instability,^47^ and this conflict has been widely studied both in prokaryotes^48^ and eukaryotes.^49^ It is revealing that we find an evidence for an evolutionary adjustment of these two fundamental levels of cellular DNA transactions—replication and gene regulation—on a purely structural level (via the embedding of the network in the chromosome), as well as on a functional level in gene expression profiles. This means that replication and transcription are coordinated from the same assessment center using the Ori–Ter axis as a system of coordinates, providing a new rationale for understanding the evolution of chromosomal gene organization.

Our results confirm previous observations of two logically distinct—digital and analog—types of transcriptional regulation in *E. coli*^20,27^ and demonstrate an anti-correlation of digital and analog control strengths across a wide range of genotypes and phenotypes. Futhermore we clearly show the dualistic nature of digital and analog control via feature selection in a classification of transcriptome profiles based on machine learning, with additional evidence for relevance of the Ori–Ter axis. This is clearly visible in Fig. 7 (RNA-Seq) and Fig. 8 (microarray), which show the correlation of each feature with analog (left) and digital (right) control strengths. First of all, the previously discussed anti-correlation of digital and analog control strengths is clearly visible. Furthermore, one can see that, indeed, the analog features tend to correlate with analog control strengths, while the digital features rather correlate with digital control strength. Only the position relative to the Ori stands out: It is (slightly) negatively correlated with both, digital and analog control strengths. These systematics of the correlations are the same for RNA-Seq data (Fig. 7) and microarray data (Fig. 8). Hence we denote this symmetry axis as a coordinator of genetic regulation. It is noteworthy that our data predominantly consist of statistical signals made visible by comparison with null models as well as methods from machine learning. In all cases we average over a wide range of conditions and individual cases (for example, the transcriptome profiles of diverse origins or the high variation in gene density across the chromosome). As a consequence of these averaging procedures most of the signals will necessarily be rather faint. We would like to emphasize, however, that each of the signals reported here is highly significant and, in combination, the collection of statistical signals from the structural investigation, the assessment of transcriptome profiles and the machine learning classification task furnish a structural and dynamical foundation of digital and analog control in bacterial gene regulation.

Taken together, these data provide a very clear picture, where the set of ideas about chromosomal DNA topology as a fundamental level embedding the transcriptional regulation in bacteria discussed in the literature already for several decades^23,24,26,27,50^ is confirmed and the evidence for an evolutionary alignment of two fundamental levels of cellular organization—replication and gene regulation—is found on a purely structural level (via the embedding of the network in the chromosome), as well as on a functional level in the design of gene expression patterns. Notably, the embedding of a network in space along a systemically defined axis can potentially be of relevance for a wide range of systems. Sensory systems for example have an axis along the hierarchical depth from the input nodes to processing nodes generating ever more abstract representations of the sensory input.^51,52^ The same is true for manufacturing systems with their hierarchy of input, production and assembly/output layers.^53^

The situation we face in our attempts to epitomize the spatiotemporal constraints imposed on the emergence of gene expression patterns is reminicent of the work by Brockmann and Helbing^54^ on the spread of epidemic diseases. They show that the complex spatiotemporal pattern of disease occurrences becomes a simple propagating wave on a tree graph derived from shortest flight distances. Distances in this “re-arranged world” are much more meaningful than geographic distances. Here, “space” is the genome and the air traffic network is the TRN, which facilitates the spreading of information across the genome. In that work^54^ the spatial distance was ignored. Adapting their formalism to the case, where a mixture of spatial and network distances defines the re-arranged “world” would allow a completely novel view on gene expression profiles.

## Methods

### Transcriptional regulatory network

In our investigation, a TRN is a graph whose nodes represent genes. If a gene *a* encodes a transcription factor *A*, which regulates another gene *b* then a link pointing from *a* to *b* is inserted in the graph. The *E. coli* TRN used for the present paper was created using data from RegulonDB,^55^ a freely available database of the regulatory network of *Escherichia coli* K-12. The TRN we have used has 1771 nodes and 3975 edges. Its minimum node degree is equal to one, the maximum node degree is equal to 496 and the average degree is equal to 4.49. The largest cluster of the TRN consists of 1678 nodes and has 3788 edges. It is a disassortative network with a degree assortativity coefficient equal to −0.32 meaning that high degree nodes tend to connect to low degree nodes and slightly avoid "hub”–"hub” connections.

It is well known that genes are organized in operons i.e., groups of genes sharing a regulatory domain. In order to check for and exclude the contributions of the “operon” effect we have also performed our investigations on a modified version of the TRN where the nodes are operons instead of genes and a link between two operons is present if a gene in one operon produces a transcription factor that regulates a gene of the second operon. The largest cluster of this operon TRN consists of 816 nodes and 1551 edges with max degree equal to 220 and average degree equal to 3.80.

### Gene proximity network

The GPN is an undirected graph of 4609 nodes and 90,878 edges. It is a formal representation of the spatial organization of the chromosome. Following the prescription from earlier work,^27^ nodes represent genes and are connected to each other, if their “centers” are separated by a distance less or equal to a distance threshold of *T*_GPN_ = 20 kilobase pairs (kbp) on the circular DNA chromosome. The info required for constructing the GPN i.e., gene names and their starting and ending positions on the *E. coli* chromosome were again obtained from RegulonDB. As a gene’s “center” position we have considered the average of its starting and ending positions.

### Transcriptome profiles and effective networks

COLOMBOS offers RNA-Seq and microarray experimental data containing differentially expressed genes between pairs of experiments. Differentially expressed genes are determined by computing the log-fold change (logFC) between the two experimental conditions. We consider three modes of differential expression depending on the logFC: “differentially regulated” (absdge) (abs(logFC) > *T*_FC_), “upregulated” (posdge) (logFC > *T*_FC_), “downregulated” (negdge) (logFC < −*T*_FC_).

We construct an effective TRN by taking the subgraph from the complete TRN consisting of all differentialy expressed genes and the links among them (see Supplementary Fig. 5 left for an example). Thus, an effective TRN is a directed subgraph of the TRN where the nodes are only the genes whose expression level has been significantly altered. Similarly, we construct an effective GPN by taking the subgraph from the complete GPN consisting of all differentialy expressed genes and the links among them. Consequently, each pair of experiments has one effective TRN and one effective GPN associated with it. In total we have analyzed 104 effective TRNs and effective GPNs from RNA-Seq data and 3969 effective TRNs and effective GPN from microarray data.

Differential expression on the gene level is translated to the operon level in the following way: an operon is considered differentially expressed, if any gene in the operon is differentially expressed. The same rule is applied for distinguishing between differentially upregulated and downregulated operons. A potentially conflicting case, where an operon consists of both, significantly upregulated and downregulated genes in the same experiment, does not occur in the data sets we analyzed.

Unless indicated otherwise, results are shown for *T*_FC_ = 2.5 and *T*_GPN_ = 20 kbp. The robustness of the results under variation of *T*_FC_ and *T*_GPN_ is demonstrated in Supplementary Figs 11–14.

### Systematic random networks

A key component of the structural part of our analysis is the arrangement of edges with respect to a given spatial axis. In order to interpret the statistics observed in the real network, we employ a simple graph-generation algorithm to create random networks with edge counts, which are systematic with respect to one predefined axis. Parameters of this algorithm are the number of nodes, *N*; the number of edges in each category, *n*(*r*_+_), *n*(*r*_−_), *n*(*l*_+_), *n*(*l*_−_), *n*(*rl*), *n*(*lr*), with respect to the chosen axis (see Fig. 1 for an illustration of the edge categories); the position of the axis, a*; the size of the genome, *g*. First, random positions for the *N* genes are created (*N*/2 per chromosomal arm). Next, random links within each category are created according to the axis a*. These networks can subsequently be analyzed via the same analysis pipeline as the real chromosomally embedded TRN.

### Graph randomization

All the results for the edge category asymmetry analysis shown here are differences between a given graph and a set of randomized graphs, serving as a null model. Here we keep the gene positions fixed and randomize the edges via switch randomization. For each randomized graph, 5000 randomization steps are performed.

### Control strengths

Qualitatively speaking, each control strength measures the agreement between a set of genes and a given network. In the induced subgraph spanned by the set of genes we compute the connectivity (specifically we compute the number of nodes with non-zero degree in the subgraph). Using a null model of randomly drawn gene sets, we then compute the *z*-score of this connectivity. This *z*-score is the control strength. Applying this procedure for the TRN yields the digital control strength; applying this procedure to the GPN yields the analog control strength.

For each effective network the control ratio *R* is calculated as the number of connected nodes *N*_connected_ (i.e., the size of the connected subnet component) over the number of isolated nodes *N*_isolated_ (i.e., the size of the unconnected subnet component), *R* = *N*_connected_/*N*_isolated_. The control type confidence, CTC^27^ or control strength, is the *z*-score of *R*, calculated from the mean *R* and its standard deviation obtained from 10,000 runs of the corresponding null model. In the case of the digital null model, the same number of affected nodes was mapped randomly on the TRN. For the analog null model, the same number of affected genes was mapped randomly on the positions in circular genome.

### Decision trees

For the decision tree implementation we choose nine features which will be the input of our machine learning model. Our decision tree model will use these features to predict whether a gene will be differentially expressed or not. These features are the following:

- PosOric = position relative to Ori.
- crp density = crp binding sites density i.e., number of cpr binding sites in a distance +/−50,000 base pairs around the gene.
- hns density = hns binding sites density i.e., number of hns binding sites in a distance +/−50,000 base pairs around the gene.
- fis density = fis binding sites density i.e., number of fis binding sites in a distance +/−50,000 base pairs around the gene.
- gpn cont = number of affected neighbors in the GPN.
- trn cont = number of affected ancestors in the TRN.
- hns dig cont = binary variable; 1, if hns is in the gene’s direct TRN predecessors, 0 otherwise.
- fis dig cont = binary variable; 1, if fis is in the gene’s TRN direct predecessors, 0 otherwise.
- crp dig cont = binary variable; 1, if crp is in the gene’s TRN direct predecessors, 0 otherwise.

In short, we assume that the differential expression of a gene is a function *f* of the above nine variables. The range of *f* is the discrete set 0, 1 where the value 1 means that the gene is differentially expressed. Thus, each of the 4602 *E. coli* genes is characterized by a nine-dimensional “vector” with the values of these nine variables as coordinates. For each of these genes the value of *f* is known (and that is true for each of the ~4000 experiments of the COLOMBOS database). Predicting the values of *f* and comparing them to the known values can be seen as a supervised learning problem. In fact, a decision tree represents a function that takes as input a vector of attribute values and returns a “decision” i.e., a single output value. A decision tree reaches its decision by performing a sequence of tests. Each internal node in the tree corresponds to a test of the value of one of the input attributes. The algorithm selects a variable and splits the data to the value of the variable that maximizes the entropy gain (or equivalently the Gini impurity gain) from the split.^56^

In our case at each node which contains for example *N* genes we calculate the value $S=-({p}_{1}{\mathrm{ln}}({p}_{1})+{p}_{0}{\mathrm{ln}}({p}_{0}))$ where *p*_1_ is the fraction of expressed genes to total genes *N* on the node and *p*_0_ is the fraction of silent genes to *N*. Then test splittings are performed and the quantity $G\,=\,(\frac{{n}_{{\mathrm{left}}}}{N}{S}_{{\mathrm{left}}}+\frac{{n}_{{\mathrm{right}}}}{N}{S}_{{\mathrm{right}}})$ is calculated. The split that maximizes the difference *S* − *G* is selected. The Gini impurity *g* = *p*_1_(1 − *p*_1_) + *p*_0_(1 − *p*_0_) is a valid and often used alternative to the entropy *S*.

Then it does the same for all other variables, finally selecting the variable and value that leads to the maximum gain among all possible choices. Thus, the main nodes split in two nodes and the process is repeated for each of them until a perfect classification is reached. Finally, we calculate the importance of each feature (variable) used for the classification process. The way to compute the feature importance values of a single tree is by traversing the tree and for each internal node that splits on feature *i* we compute the error reduction of that node multiplied by the number of samples that were routed to the node and sum this quantity for all nodes to estimate the feature importance of variable *i*. The error reduction depends on the impurity criterion that you use (Gini or entropy). It is the impurity of the set of examples that gets routed to the internal node minus the sum of the impurities of the two partitions created by the split. This is the way that regression trees are implemented in scikit-learn^57^ which is rapidly becoming a standard machine learning tool.

### Reporting summary

Further information on research design is available in the Nature Research Reporting Summary linked to this article.

## Supplementary information

Supplementary Information

Reporting Summary

## Data Availability

All data analysed during this study are publicly available via the databases referenced in the article.

## Code availability

The code used to perform the analyses presented in the current study is available from the corresponding author on reasonable request.
