# Supplementary material for: Chromosomal origin of replication coordinates logically distinct types of bacterial genetic regulation
Source: NPJ Syst Biol Appl. 2020 Feb 17;6:5. doi: 10.1038/s41540-020-0124-1 (PMC7026169; doi:10.1038/s41540-020-0124-1)
Supplement: Supplementary file 1 — Supplementary Information [file 41540_2020_124_MOESM1_ESM.pdf]

Supplementary Information for:
Chromosomal origin of replication coordinates logically distinct types of bacterial genetic regulation

[1,2*]Kosmas Kosmidis [3] Kim Philipp Jablonski
[4] Georgi Muskhelishvili [3] Marc-Thorsten Hütt

[1] Division of Theoretical Physics, Physics Department, Aristotle University of Thessaloniki, 54124 Thessaloniki, Greece
[2] PharmaInformatics Unit, Research Center ATHENA, Athens, Greece
[3] Department of Life Sciences and Chemistry, Jacobs University, Bremen, Germany
[4] Department of Biology, Agricultural University of Georgia, Tbilisi, Georgia

Supplementary Discussion: Spatial and topological distances

Here we summarize the analysis of the chromosomal embedding of the transcriptional regulatory network (TRN) using standard tools from network science. An extended version of this analysis has been published in Kosmidis and Hütt (2019).
A statistical analysis of distances and link densities following the standard methodology for spatially embedded networks (e.g., Kosmidis et al. (2008)) already reveals some key features of the chromosomal embedding of the TRN. The overarching questions is: How does the density of connections or their ranges change with spatial distance?
The study the TRN of *E. coli* as a spatially embedded network primarily consists of understanding the interconnection of the topological distance l (number of "hops") between two nodes, which is a standard network property, and the spatial distance r between the same nodes. The TRN nodes are *E. coli* genes and they are located on a single circular DNA molecule (the chromosome). We define the spatial distance between two nodes as the minimum number of base pairs separating the centers of these genes. Of course, the *E. coli* genes are actually embedded in the normal 3D space and hence other distances between genes can be defined, the normal Euclidean distance being the most obvious example. However, this Euclidean distance is dynamic depending on the chromosomal conformation at each time step and hence impractical as a choice. In what follows the term spatial distance refers to the, constant in time, number of base pairs that separate the two genes. It should be noted, however, that nodes having a large spatial distance may in fact be in physical proximity due to the conformation of the DNA molecule, especially when the DNA supercoiling is high.
Figure 1 shows the mean spatial distance r (in base pairs) of the TRN genes as a function of the mean topological distance l. A dotted horizontal line marking the mean spatial distance of all *E. coli* genes (included and non-included in the TRN) is shown for comparison. Only the genes that are part of the largest cluster (trn_lc) of the TRN are used since the topological distance of nodes belonging in different clusters is not well defined. We observe that nodes that are directly connected in the TRN, i.e. at $l = 1$ have a shorter mean spatial distance r. This is partly, but not completely, due to the operon effect. We have verified, that if we transform the TRN from a gene-node network to an operon-node network this decreased value for r at $l = 1$ remains indicating that the effect cannot be explained solely from the tendency of genes to form operons and that there must be additional organizational reasons that promote gene spatial proximity beyond the operon level. We can also see that also nodes at $l = 2$, i.e. second neighbors in the TRN, tend to be closer that the average while the few nodes that are at the largest topological distance $l = 9$ at the TRN have indeed a large spatial distance on the *E. coli* chromosome.

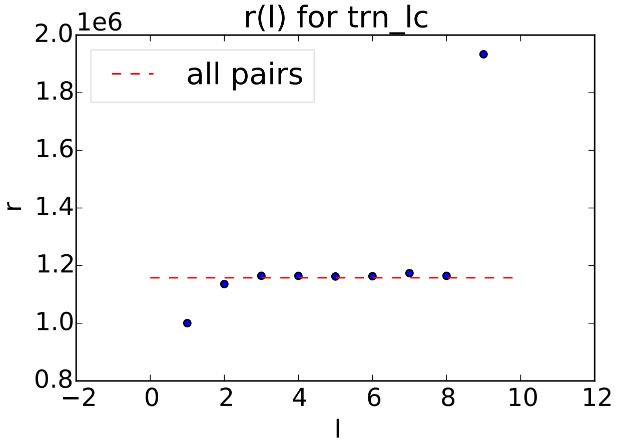

Figure 1: Mean pairwise distance r (in base pairs) of the TRN genes as a function of the mean topological distance l. The dotted horizontal line is the mean pairwise distance of all *E. coli* genes (included and non-included in the TRN). Figure adapted from Kosmidis and Hütt (2019).

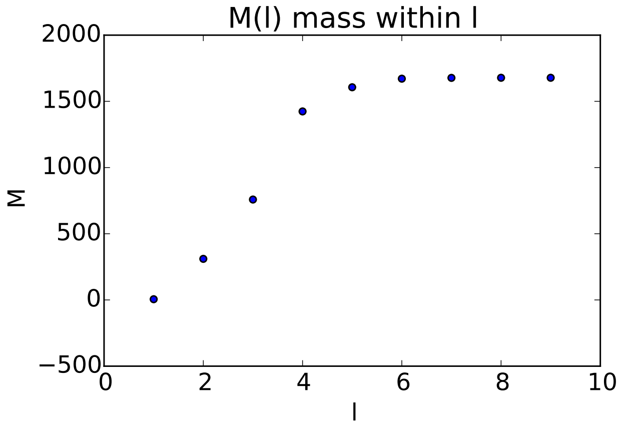

Figure 2: "Mass" (i.e. mean number of nodes) within topological distance l for the *E. coli* TRN. Figure adapted from Kosmidis and Hütt (2019).

Figure 2 shows the mean number of nodes ("mass") M within topological distance l. This quantity gives some insight on the network structure. For a linear chain for example M is proportional to l while for a "tree" structure M increases exponentially with l. In Fig. 2 we observe an initially sharp increase followed by a plateau. This is consistent with a network that has a capable number of "hubs" connected to some more or less linear "dangling" ends. The sharp increase is due to the presence of the "hubs". As soon as a hub is reached a large number of nodes becomes immediately accessible at the next topological step. Of course a saturation effect is to be expected because of the finite size of the network, hence the plateau.

Supplementary Methods: EDURA for systematic random networks

The question, whether the links in a spatially embedded network are non-random with respect to a given spatial axis, requires a dedicated statistical analysis method. This is the EDURA method. A schematic diagram of the EDURA method is given in Figure 3).
Here we illustrate the EDURA method using systematic random networks (see **Methods** in the main text), which contains a $\pm$ asymmetry with respect to a specific axis, and performing an edge category asymmetry analysis (computing $A^r_\pm$, $A^l_\pm$ and $A_{\leftrightarrow\updownarrow}$ and then studying the correlations among these asymmetries). Figure 4 summarizes the main result.
We performed a wide range of analyses with such systematic random networks, all of which showed the capability of the EDURA method to detect the axis associated with the systematic signals.

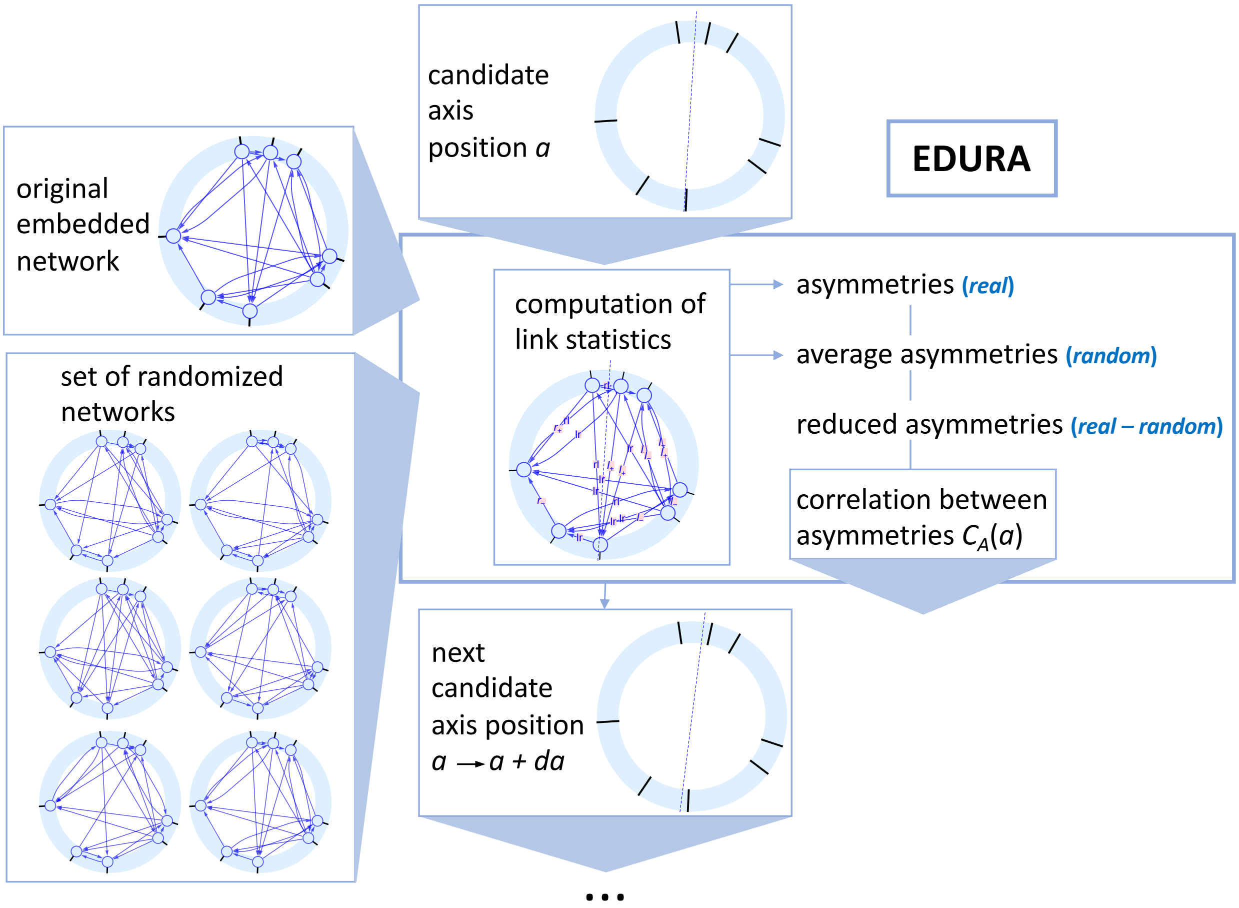

Figure 3: Schematic representation of our new analysis method for spatially embedded networks. The EDURA method (EDURA = Edge Distribution Under Rotation of an Axis) starts by assuming the position a of a spatial axis in a given spatially embedded network and then evaluating *link statistics* with respect to this axis (thus determining the numbers of links in the domain of the axis pointing in forward direction, l_+, in backward direction, l_-, or towards the right domain, lr, etc.). From these data, *asymmetries* (within-domain links vs. across-domain links, forward vs. backward, etc.) are computed. These data are contrasted with the corresponding information from switch-randomized networks serving as null model, leading to *reduced asymmetries*. The output signal of EDURA is then the correlation $C_A(a)$ of reduced asymmetries. Once this step is completed, the axis position is advanced, $a \to a + da$. In this way, axis positions with non-random link distributions can be detected.

Supplementary Discussion: Digital and analog control

As an example of this analysis, the computation of effective networks and the statistical signal extracted from them, is given in the supplementary information.

In order to understand the importance of the two distinct control mechanisms that are possibly participating in gene regulation we follow the methodology proposed in Marr et al. (2008) and calculate the digital control strength (digital control-type confidence, digitalCTC) and analog control strength (analog control-type confidence, analogCTC). Definitions and details are presented in the **Methods** section under the subsection **Control Strengths**. Figures 6 and 7 show two examples of data sets exhibiting high values of digital and analog control. Figure 6 shows the digitalCTC associated to the effective TRN of differentially expressed genes for contrasts GSM256904_ch1 and GSM256868_ch1 again from the GSE10158 experiment. The histogram shows the randomized distribution of the connected to isolated nodes ratio R (see **Methods**) while the dashed vertical line the R for the actual effective TRN. The distribution is used to convert R to a z-score resulting in a digitalCTC of 14.80.

Figure 7 shows the analogCTC associated to the effective GPN of differentially expressed genes for contrasts

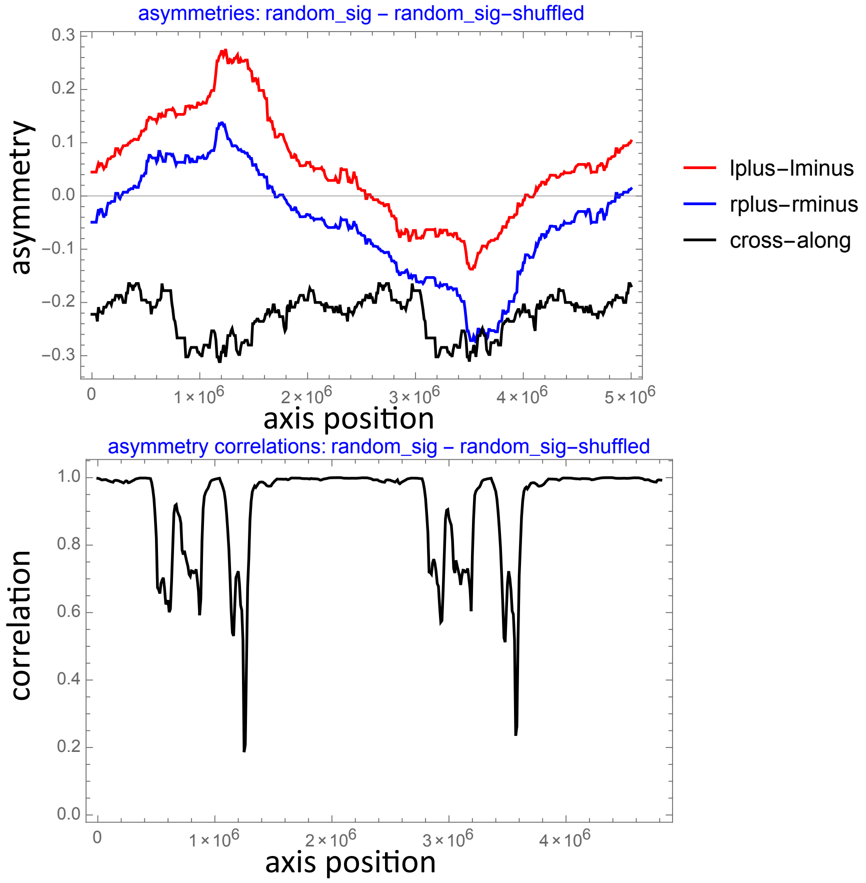

Figure 4: Edge category asymmetry analysis for a random systematic network generated to contain a strong $\pm$ asymmetry with respect to an axis located at 1 Mbp. Parameters for the network generation are: $N = 200$, $n(r_+) = 200$, $n(r_-) = 400$, $n(l_+) = 200$, $n(l_-) = 400$, $n(rl) = 300$, $n(lr) = 300$, $a^* = 1$ Mbp and $g = 5$ Mbp. (A) Asymmetries as a function of the assumed axis position. (B) Correlation coefficient of $A^r_\pm$ and $A^l_\pm$ as a function of the assumed axis position.

GSM1099469_ch2 versus GSM1099469_ch1 from the GSE45228 experiment. This experiment is described in COLOMBOS as "Exposure of Bacillus VOCs vs. Control". The histogram shows the randomized distribution of the connected to isolated nodes ratio R (see **Methods**) while the dashed vertical line the R for the actual effective GPN. The distribution is used to convert R to a z-score resulting in a analogCTC of 28.47.

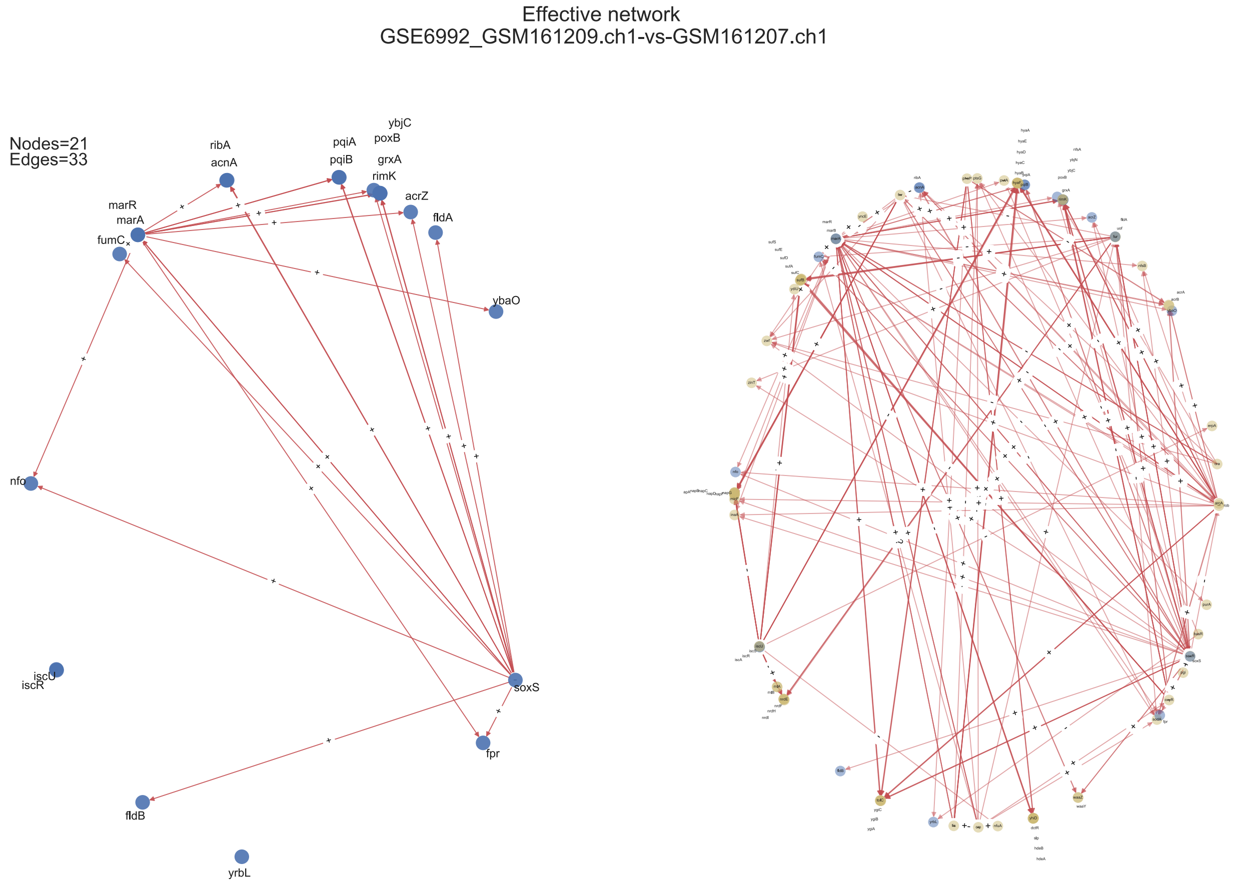

Figure 5: Example of an effective TRN network. (Left) The 22 genes whose expression level was significantly altered comparing contrasts with id's GSM256904_ch1 and GSM256868_ch1 from experiment GSE10158 from the COLOMBOS database. (Right) An "extended" TRN subgraph which contains the differentially expressed genes (blue points) plus all the genes that are connected to the differentially expressed ones in the *E. coli* TRN although without significantly altered expression levels (yellow points). Genes are positioned on a circle in accordance to their position on the *E. coli* chromosome.

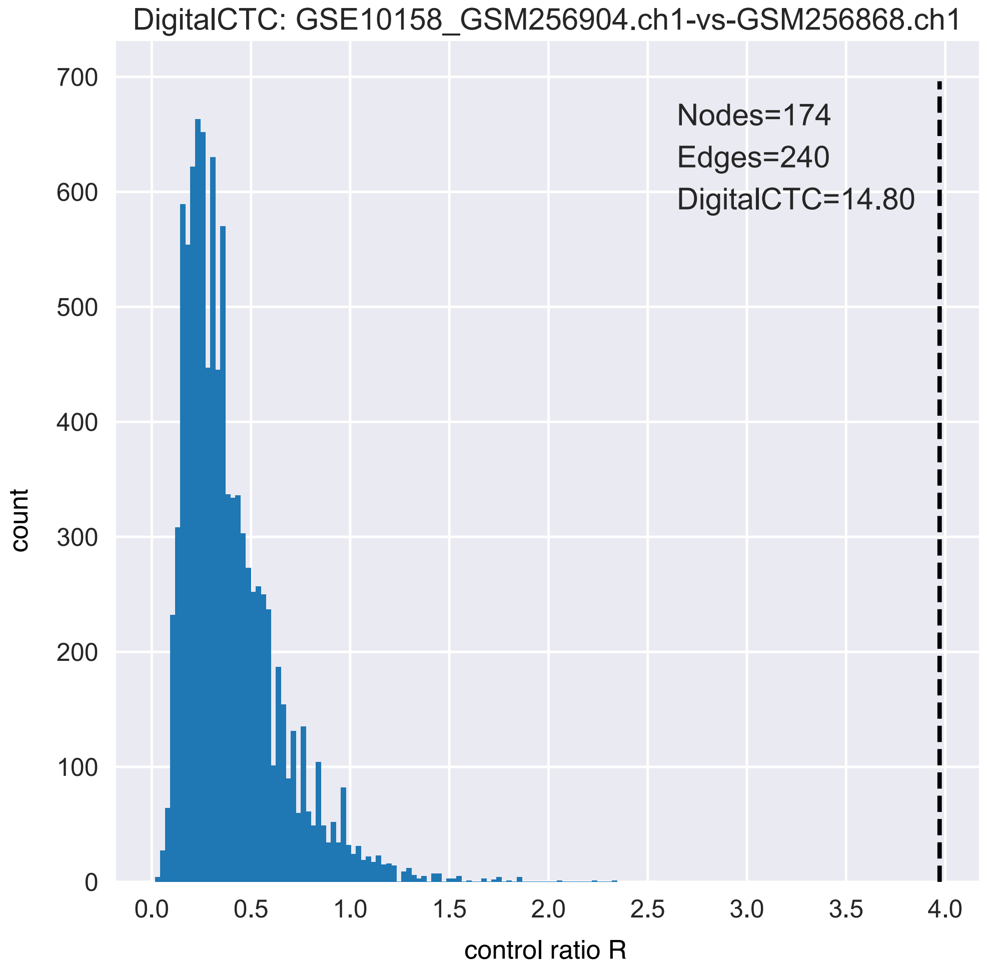

Figure 6: Example of high digital control strength. Effective TRN of differentially expressed genes for contrasts GSM256904_ch1 and GSM256868_ch1 from the GSE10158 experiment. Histogram of the randomized distribution of the connected to isolated nodes ratio (or 'control ratio'; see Methods in the main text) R. The dashed line shows the R of the actual effective TRN.

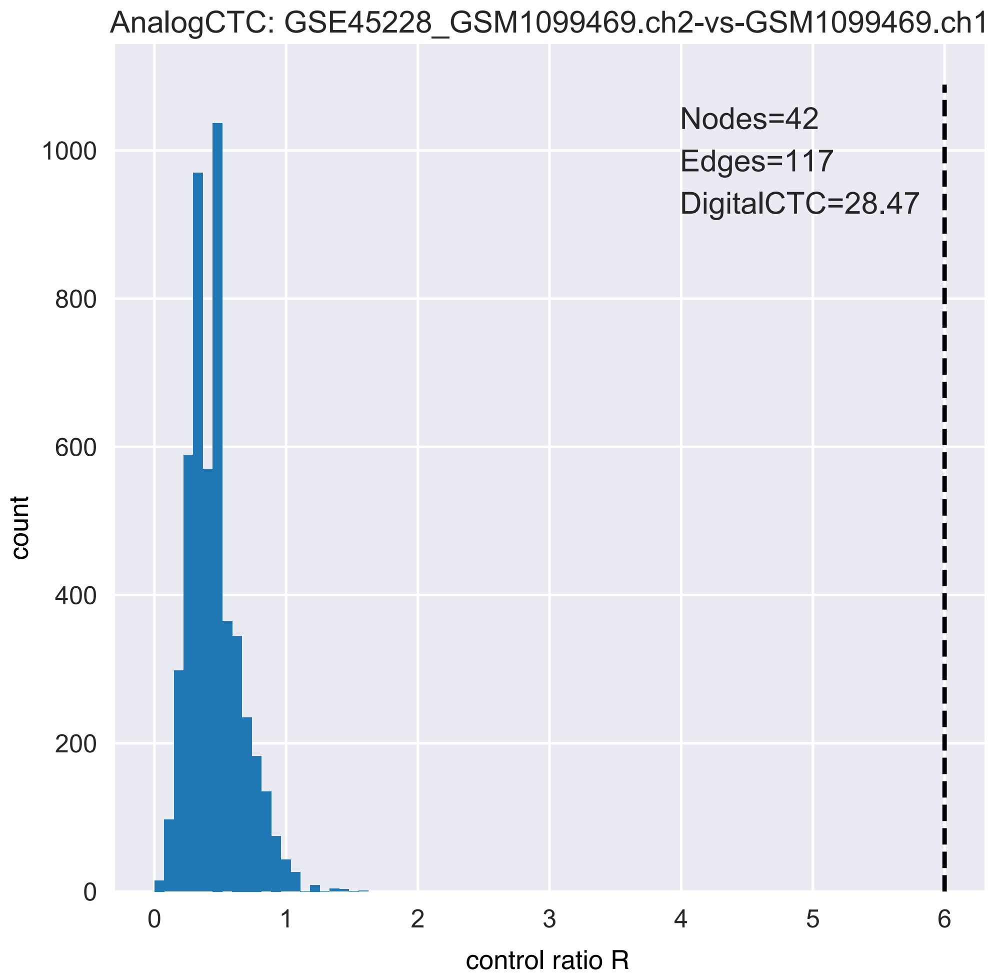

Figure 7: Example of high analog control strength. Effective GPN of differentially expressed genes for "contrasts"GSM1099469_ch2 versus GSM1099469_ch1 from the GSE45228 experiment. Histogram of the randomized distribution of the connected to isolated nodes ratio (or 'control ratio'; see Methods in the main text) R. The dashed line shows the R of the actual effective GPN.

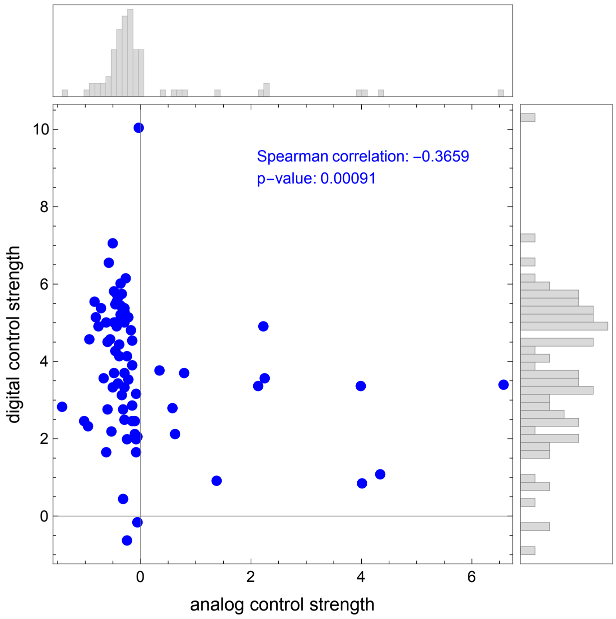

Figure 8: Digital vs analog control strengths for gene-level RNA-Seq data (negative expression changes). Scatter plot of digitalCTC versus analogCTC based on effective networks derived from down-regulated genes only.

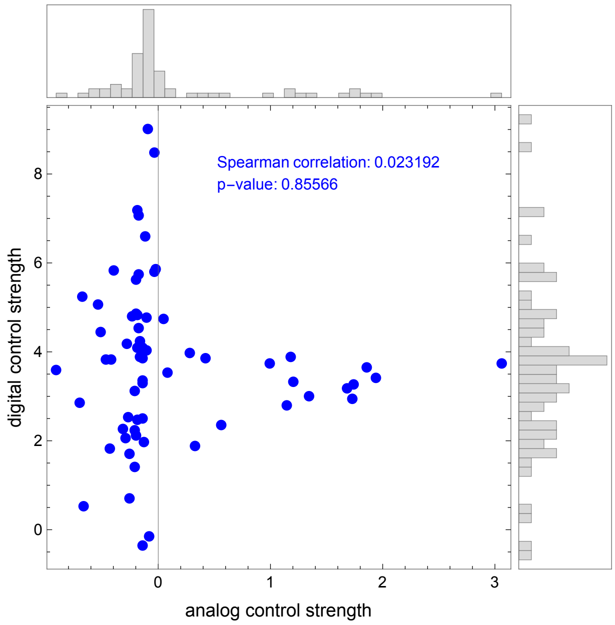

Figure 9: Digital vs analog control strengths for gene-level RNA-Seq data (positive expression changes). Scatter plot of digitalCTC versus analogCTC based on effective networks derived from up-regulated genes only.

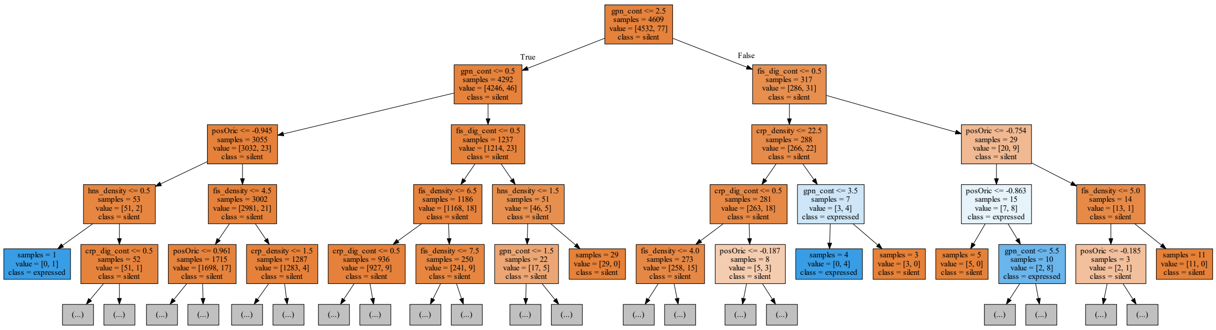

Figure 10: Decision Tree for the differential expression pattern between contrasts GSM991204_ch1 versus GSM991216_ch1 from the RNAseq experiment GSE40313. Each node is splitted to the variable and value that maximizes the impurity gain. The tree is used to estimate the importance of each of the nine features described in **Methods-Decision Trees**.

Supplementary Discussion: Decision tree analysis

We describe each *E. coli* gene by a "vector" of 9 features. These are described in detail in **Methods** in the **Decision Tree** subsection. Among these crp density, hns density and fis density and gpn cont are mainly analog control features while trn cont, hns dig cont, fis dig cont and crp dig cont are mainly digital control features. The position relative to the origin of replication (posOric) was included as a feature and was observed that without it no method of classification could achieve a perfect classification score. As a target outcome, for each of the effective networks constructed from the COLOMBOS database, we have assigned the value 0 to a gene not significantly differentially expressed (i.e. not in the corresponding effective network) and the value 1 otherwise. We have used Decision Trees in order to have an estimation of the importance of each feature in the task of accurate classification of the target outcome. This method was selected since none of the simple – linear – classification methods has obtained an acceptable classification score due to the complexity of the problem.

In Fig. 10 we show an example of a decision tree used to classify the differential expression pattern between contrasts GSM991204_ch1 versus GSM991216_ch1 from the RNAseq experiment GSE40313 whose description is "Genomic analysis of the combined effects of H-NS and co-regulators on *E. coli* gene expression". We see that we start with a set that contains 4609 "samples"(genes) among whom 4532 were silent (not differentially expressed) while 77 were expressed. The largest reduction in entropy is achieved by using the feature "gpn cont" and the value 2.5 as threshold. This action splits the "samples" in two subgroups. The first one contains 4292 genes of whom 4246 silent and 46 expressed genes. The second contains 317 genes of whom 286 silent and 31 expressed genes. The fraction of expressed genes on the initial set is $p_1 = 77/4609$ and $p_0 = 77/4609$. This lead to an entropy $S = -(p_1 \ln(p_1) + p_0 \ln(p_0)) = 0.0849$. The splitting in these two subgroups leads to a value $G = (\frac{n_{left}}{N} S_{left} + \frac{n_{right}}{N} S_{right}) = 0.0772$ and it is the split that achieves the largest entropy reduction. The process is repeated for each node until a perfect classification is achieved i.e the initial set is divided in nodes that contain only silent or only expressed genes (see also **Methods-Decision Trees** in the main text).

Supplementary Figures: Results for different values of the fold change threshold and the GPN distance threshold

Figures 11–14 show the main result of the functional analysis (i.e., the control strengths for the effective networks derived from gene expression data) as a function of the log fold-change (logFC) threshold for different values of the GPN distance threshold.

- Figure 11: gene-level RNA-Seq data
- Figure 12: gene-level microarray data
- Figure 13: operon-level RNA-Seq data
- Figure 14: operon-level microarray data

In this way, these figures allow us to confirm the validity of this key finding – the pronounced anti-correlation of digital and analog control – over a wide range of the two main parameters of our analysis.

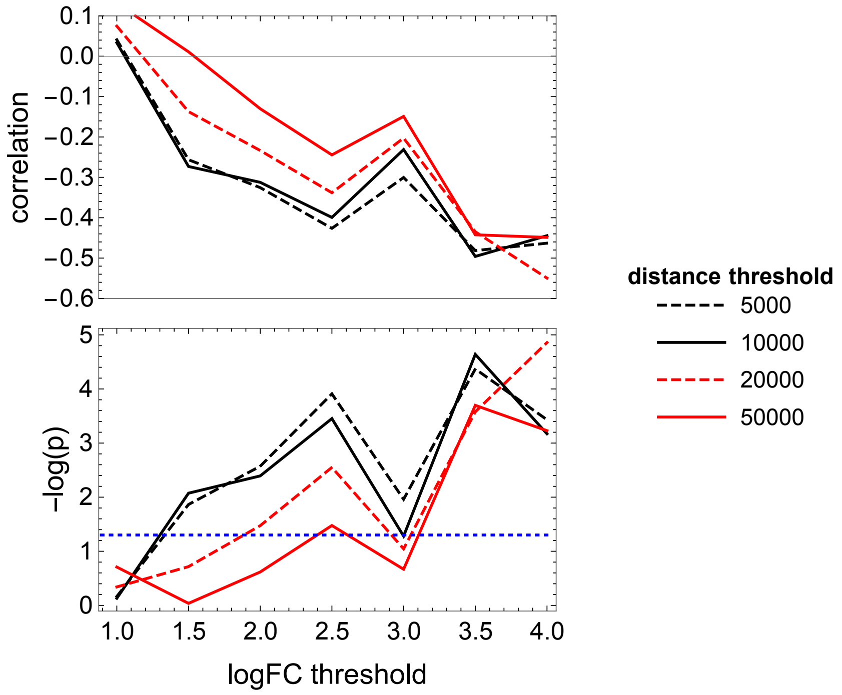

Figure 11: Spearman correlation coefficient between digital and analog control strengths (upper panel) and the negative logarithm of the corresponding p-value (lower panel) as a function of the logFC threshold for different values of the GPN distance threshold (dashed black line: 5 kbp, full black line: 10 kbp, dashed red line: 20 kbp, full red line: 50 kbp). The dotted blue line in the lower panel indicates a significance threshold of 0.05 (note the negative logarithmic scale: higher values = more significant). Differential expression has been derived from gene-level RNA-Seq data.

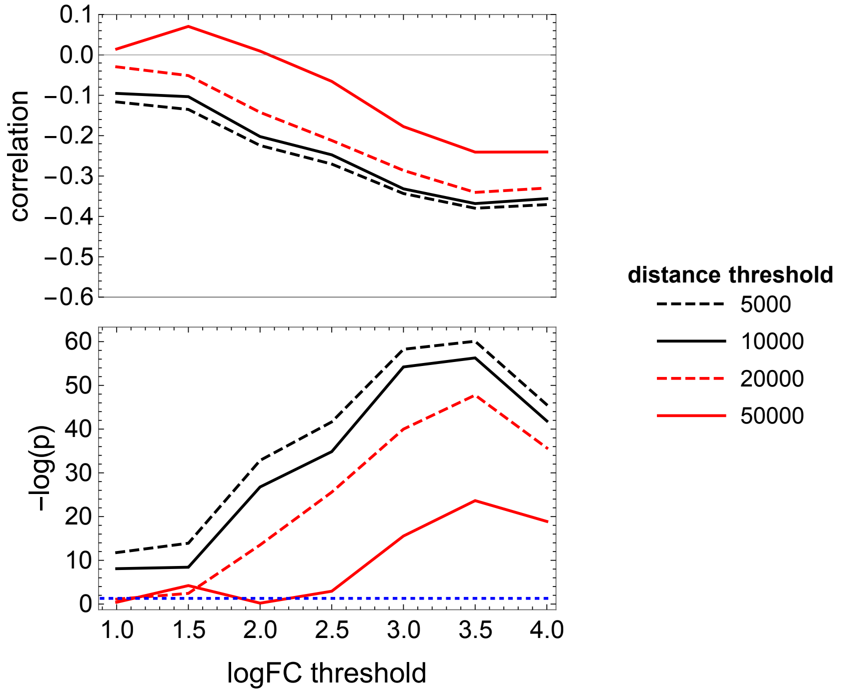

Figure 12: Same as Figure 11, but differential expression has been derived from gene-level microarray data.

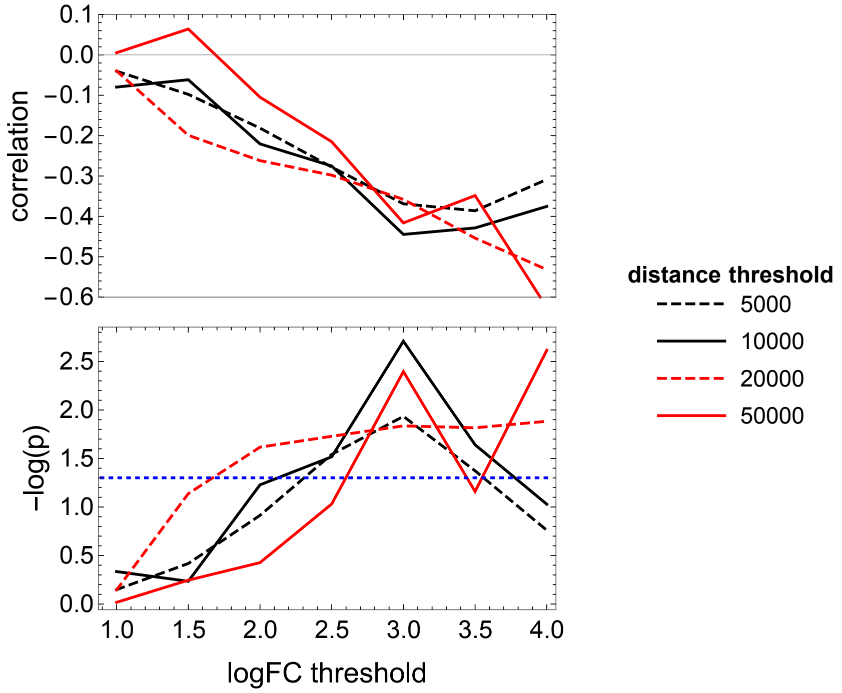

Figure 13: Same as Figure 11, but differential expression has been derived from operon-level RNA-Seq data.

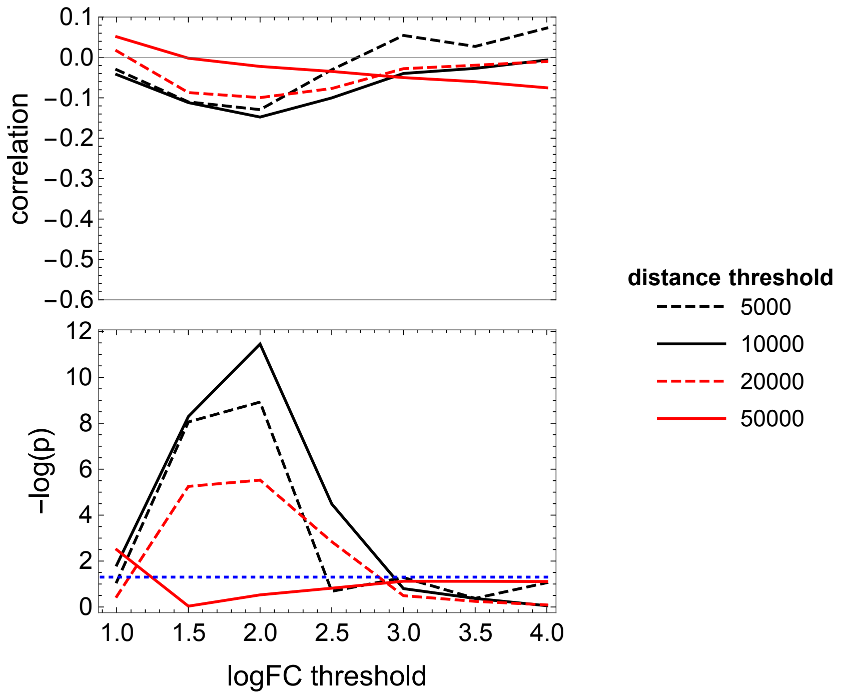

Figure 14: Same as Figure 11, but differential expression has been derived from operon-level microarray data.

Supplementary Figures: Heatmap representations of the scatter plots in Figures 5 and 6 in the main text

The visual inspection of the scatter plots in Figures 5 and 6 in the main text is challenging due to the non-Gaussian distribution of the digital and analog control strengths. An alternative representation is obtained by substituting the control strength values by their respective rank and then performing local averages in this rank-based scatter plot. These smoothed density historgrams (or 'heatmaps') are shown in Figures 15 and 16 for the RNA-Seq data and the microarray data respectively. For the RNA-Seq data, the buffering relationship between digital and analog control is clearly discernible here: High rank values of analog control go along with low rank values of digital control and *vice versa* (cf. the two red peaks in Figure 15). The heatmap for the ranked digital and analog control strenths for the microarray data is displayed in Figure 16. Again, the buffering relationship between digital and analog control is the dominant feature of this plot.

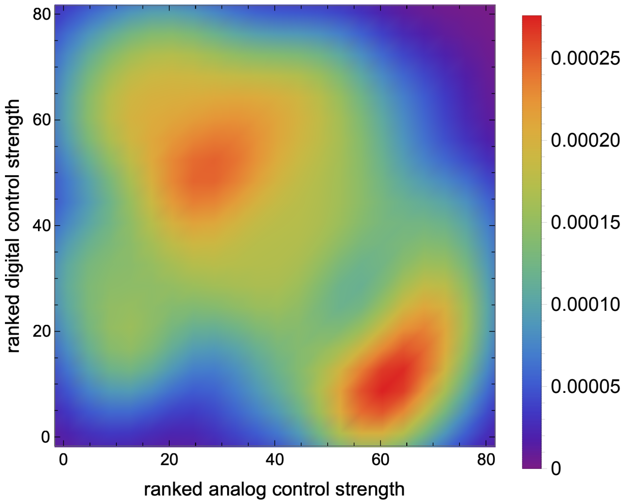

Figure 15: Smoothed density histogram ('heatmap') derived from the scatter plot of digital control strengths vs. analog control strengths for the RNA-Seq contrasts. Parameter values are the same as in Figure 5 of the main text.

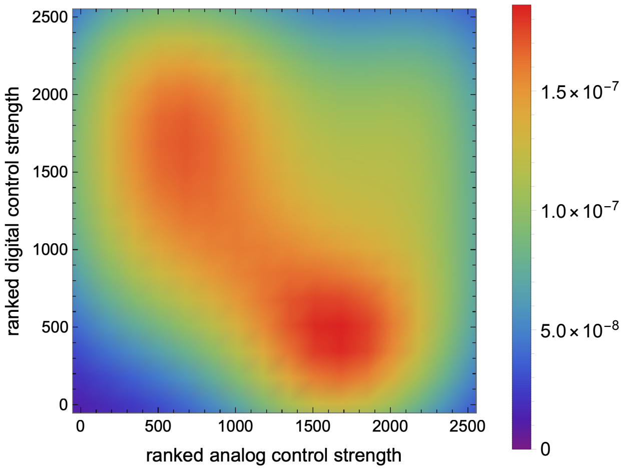

Figure 16: Smoothed density histogram ('heatmap') derived from the scatter plot of digital control strengths vs. analog control strengths for the microarray contrasts. Parameter values are the same as in Figure 6 of the main text.

Supplementary References

Kosmidis, K., Havlin, S., and Bunde, A. (2008). Structural properties of spatially embedded networks. *EPL (Europhysics Letters)*, 82(4):48005.

Kosmidis, K. and Hütt, M.-T. (2019). The *E. coli* transcriptional regulatory network and its spatial embedding. *The European Physical Journal E*, 42(3):30.

Marr, C., Geertz, M., Hütt, M.-T., and Muskhelishvili, G. (2008). Dissecting the logical types of network control in gene expression profiles. *BMC systems biology*, 2(1):18.
